# Attention bias to threat indicates anxiety differences in sheep

**DOI:** 10.1098/rsbl.2015.0977

**Published:** 2016-06

**Authors:** Caroline Lee, Else Verbeek, Rebecca Doyle, Melissa Bateson

**Affiliations:** 1 CSIRO, Agriculture, Armidale, Australia; 2 Animal Welfare Science Centre, University of Melbourne, Melbourne, Australia; 3 Centre for Behaviour and Evolution, Newcastle University, Newcastle upon Tyne, UK

**Keywords:** affective states, animal welfare, threat perception, vigilance

## Abstract

Humans and animals show increased attention towards threatening stimuli when they are in increased states of anxiety. The few animal studies that have examined this phenomenon, known as attention bias, have applied environmental manipulations to induce anxiety but the effects of drug-induced anxiety levels on attention bias have not been demonstrated. Here, we present an attention bias test to identify high and low anxiety states in sheep using pharmacological manipulation. Increased anxiety was induced using 1-methyl-chlorophenylpiperazine (m-CPP) and decreased anxiety with diazepam, and then we examined the behaviour of sheep in response to the presence of a dog as a threat. Increased attention towards the threat and increased vigilance were shown in sheep that received the m-CPP and reduced in sheep receiving the diazepam. The modulated attention towards a threat displayed by the m-CPP and diazepam animals suggests that attention bias can assess different levels of anxiety in sheep. Measuring attention bias has the potential to improve animal welfare assessment protocols.

## 1. Introduction

Cognitive methods for assessing affective states in animals are increasingly being used by researchers interested in measuring and improving animal welfare. Judgement biases, in which individuals interpret ambiguous cues more positively or negatively depending on their affective states, have been widely reported in a range of animal species [1]. However, the assessment of judgement bias typically requires extensive training and is impractical in applied contexts. Attentional biases, in which anxious individuals show an increased tendency to direct their attention towards threatening stimuli, potentially offer a faster method for assessing certain types of affective state. While a link between negative affect (specifically anxiety) and attention biases is well established in humans [2], attention bias has thus far received limited study in non-human animals. In support of the approach, rhesus macaques (*Maccaca mulatta*) altered their vigilance towards aggressive faces when subjected to stressful procedures and a period of enrichment [3]. In another study, starlings (*Sturnus vulgaris*) were more vigilant and had decreased willingness to feed following playback of an alarm call when they had been deprived of water bathing necessary for feather maintenance [4]. Hence, there is limited evidence that assessment of attentional responses to a threat provides insight into the anxiety levels of non-human animals. A strong validation of the approach requires demonstrating that drugs known to increase or decrease anxiety levels produce the predicted effects on attentional biases.

The aim of this study was to develop an attentional bias task for sheep, an agricultural species for which there are a range of welfare concerns [5], and to validate this with anxiogenic and anxiolytic drugs. Our attentional bias task for sheep was inspired by the starling study described above involving an arena, a food source and the response to a known source of threat (a live dog). To validate the test, we pharmacologically induced high and low anxiety states in the sheep using anxiogenic (methyl-chlorophenylpiperazine (m-CPP)) and anxiolytic (diazepam) drugs. We predicted that sheep receiving the anxiogenic drug would be more vigilant, show more attention towards the threat, and be less willing to feed after exposure to the dog, whereas sheep receiving the anxiolytic drug would demonstrate converse responses.

## 2. Material and methods

This experiment used 60 2-year-old female Merino sheep (averaging 40.4 ± 0.5 kg). For one week prior to testing, the sheep were kept in three equal groups to facilitate familiarization with eating pellets from 10 buckets per group.

Sheep were randomly allocated to one of three treatments (*n* = 20 per treatment): (i) control (receiving saline i.m.), (ii) anxiolytic (diazepam, 0.1 mg kg^−1^ i.v.) and (iii) anxiogenic (m-CPP, 2 mg kg^−1^ i.m.). This dose of diazepam has been used previously in sheep and induces positive affect without signs of sedation [6]. Methyl-chlorophenylpiperazine is a serotonin agonist psychoactive drug that has been reported to induce anxiety in a range of species [7]. The dose has been used previously in sheep, and increases anxiety without adverse effects on locomotion [8,9]. A lower dose of m-CPP (1 mg kg^−1^ i.m.) was shown to induce anxiety in younger sheep [7]. Each sheep was injected 30 min before testing with their allocated drug or saline treatment. This timing was selected as maximum concentrations in serum were reported in sheep 14.6 min after i.m. injection with diazepam and levels were maintained for up to 1 h [10].

**Figure 1.**
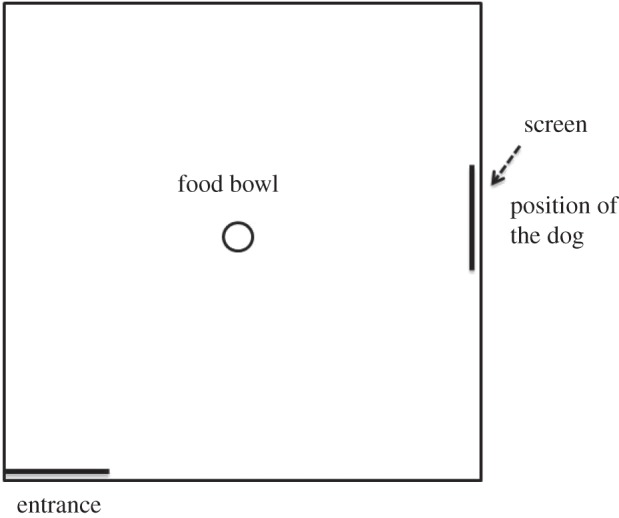
The threat perception test arena (4 × 4 m) with the familiar food bowl placed in the centre of the arena. The dog was visible for the first 10 s of the test. Figure is not to scale.

On the test day, sheep were bought into the yards from their home paddocks. The attention bias test arena (4 × 4 m) was enclosed so that animals could not see outside (figure 1). In the centre of the arena, a feed reward (200 g) was placed in a familiar bucket. Individual sheep entered the arena for 190 s and were tested in random order. A dog sitting quietly outside the arena was visible through a window on the side of the arena. After 10 s, the window was closed and the dog removed to a waiting area located 20 m away. Video cameras recorded the sheep to measure response to the dog (freezing behaviour, time spent looking at the dog), attention towards the threat, vigilance behaviour, zones crossed and latency to feed. Vigilance was defined as the head at shoulder height or higher. Attention towards the threat was defined as time spent looking in the direction of the closed window during a 60 s period immediately following removal of the dog. Zones crossed was the number of squares entered when the arena was divided into nine equal squares. The number of vocalizations was measured by a person outside the arena. The person recording the behaviour was blind to the treatments. At the cessation of testing, sheep were returned to the paddock.

Data were analysed in R v. 3.2 [11]. Attention towards the threat was analysed using a linear model fitting the treatment effect. Vigilance, zones crossed and vocalization data were analysed by the Kruskal–Wallis non-parametric test as they were not normally distributed and could not be improved by transformation. Statistical differences were investigated using post hoc multiple comparison tests using the package pgirmess [12]. Latency to feed was analysed with Cox's proportional hazards model using survival analysis [13] as a number of sheep failed to feed within 190 s. This was deemed as a censored result and was recorded as a ‘survival’ incidence. A hazard ratio of more than 1 indicates a higher likelihood of feeding and values between 0 and 1 indicate a lower likelihood.

## 3. Results

Fifty-nine of the 60 sheep demonstrated freezing behaviour in response to the presence of the dog. In the 10 s period when the dog was shown, there were no treatment differences in the time sheep spent vigilant (average of 9.2 s; *p* = 0.11) or looking at the dog (7.8 s; *p* = 0.95). Following removal of the dog (table 1), attention towards the threat was significantly affected by treatment (*p* < 0.01), with the diazepam group showing the lowest attention, followed by the control group, while the m-CPP group paid most attention towards the threat. The total duration of vigilance was significantly affected by treatment, with the diazepam group being the least vigilant, then the control group, while the m-CCP group were most vigilant. Post hoc tests indicated that the m-CPP group were significantly more vigilant than the diazepam group (observed difference 14.0 > critical difference 13.2). The control was not significantly different from either m-CPP (observed 5.2 < critical 13.2) or diazepam groups (observed 8.8 < critical 13.0). There was no effect of treatment on zones crossed or vocalizations.

**Table 1.** Mean (±s.e.m.) responses of the sheep following removal of the dog. ^abc^Different superscripts within rows indicate a significant difference between treatments.

behavioural measure	diazepam	control	m-CPP	*p*-value	*H*-value
attention towards the threat (s)	14.2 ± 1.65^a^	21.5 ± 1.66^b^	39.4 ± 1.70^c^	<0.01	
duration vigilant (s)	154 ± 5^a^	164 ± 3^a,b^	170 ± 1^b^	<0.05	
mean rank duration vigilant (s)	22.5 ± 3.84^a^	31.3 ± 3.95^a,b^	36.5 ± 3.24^b^	0.036	6.66
mean rank zones crossed	26.58 ± 4.11	26.75 ± 3.65	37.03 ± 3.52	0.1	4.69
mean rank number of vocalizations	27.38 ± 2.67	33.92 ± 3.26	28.63 ± 2.85	0.25	2.79

**Figure 2.**
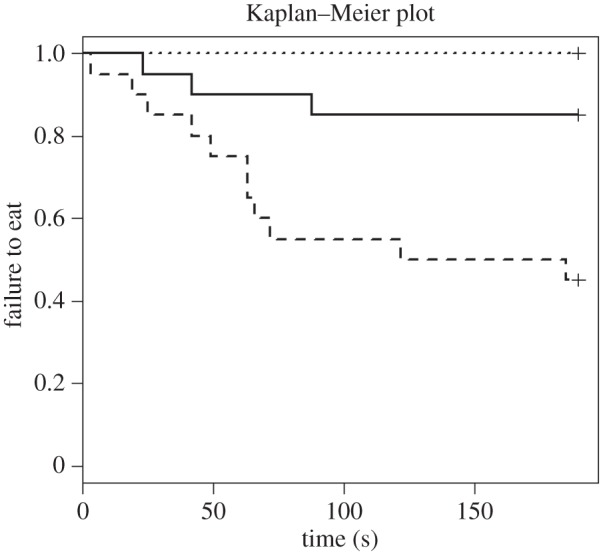
Kaplan–Meier curves for the attention bias test; solid lines, control; dashed lines, DZP; dotted lines, m-CPP. Each time a sheep commenced feeding, the probability on the *y*-axis drops.

With a hazard ratio of 4.64, the diazepam group was significantly more likely to feed than the control group (*p* = 0.01, figure 2); a total of 45% of sheep from this group failed to eat and the median latency to feed was 153 s. The hazard ratio for the m-CPP sheep was 0, indicating that none fed. There was no statistically significant difference between the m-CPP and the control group (*p* = 0.98), and 85% of the control sheep failed to eat. Owing to the low feeding rate, no median exists for either of these groups.

## 4. Discussion

The drug treatments altered attentional-orienting towards threats as predicted, which provides, to our knowledge, the first evidence of pharmacological validation of a test for attention bias in animals. Almost all of the sheep responded to the dog by being vigilant, looking at the dog and freezing. Indeed, freezing and looking towards a threat have been reported as indicators of fear in sheep [14], which suggests that the sheep were fearful and did perceive the dog as a threat. As we did not include a treatment without exposure to the dog, we are unable to distinguish the effect of the drug treatment in isolation from the response of a treated animal to the threat. Future study designs should include a treatment without the dog. Similar to human studies [2], attention towards a threat and vigilance were our key measures of attentional-orienting, which were shown to be higher in the m-CPP-treated sheep and lower in the diazepam group. This response is in agreement with the reduced vigilance response shown by sheep that received diazepam when subjected to an isolation test [6]. Attention towards a threat and vigilance were shown to increase in rhesus macaques when they were shown aggressive faces and this was used to measure social attentional bias [15]. Vigilance was also used as a measure of attentional-orienting in starlings that were denied access to water bathing and were found to be more vigilant in response to an alarm call than those that were able to water bathe [4]. These differences were interpreted to indicate higher anxiety levels in birds denied access to water bathing. Our study is, we believe, the first to pharmacologically induce different states of anxiety and show that attention bias to threat is increased in animals in high anxiety states and decreased in low anxiety states.

The use of pharmacological models to induce positive and negative affective states has advantages in terms of enabling standardized administration with appropriate controls, and drugs remain active for the duration of the test, which are more difficult to manage with an environmental treatment. There are also limitations with using pharmacological manipulations to induce anxious states in animals, including that the drugs may directly affect the measures recorded. We found no differences between treatments in the general activity of sheep (zones crossed and vocalizations) [16], which suggests that diazepam did not have any obvious sedative effects and that attention and vigilance differences were not explained by differences in general activity. However, feeding motivation has been reported to be reduced in rats receiving m-CPP [17], while diazepam was shown to increase feed intake in sheep [6]. In the starling study [4], latency to feed following the alarm call was increased in birds denied access to water bathing compared with birds that were able to water bathe, indicating that birds were more cautious and less willing to feed. In this study, there were no differences between m-CPP and the control treatment in their latency to eat, but diazepam-treated sheep were more willing to feed following exposure to the threat. Similar findings were reported in sheep in a feeding motivation test, with no differences between m-CPP and a control group but differences between m-CPP and diazepam [7]. However, because of the known effects of the drug treatments on feeding behaviour, we are not able to differentiate between differences in latency to feed due to the animals' response to the threat itself and the drug treatments directly affecting feeding behaviour. Nonetheless, the key measures of attentional-orientation: attention towards a threat and vigilance, were altered in response to the pharmacological treatments, which suggests that the attention bias test is measuring differences in anxiety states in sheep. Development of the attention bias method provides a more rapid test of affective states compared with judgement bias methods which require significant prior training. This may support more practical welfare assessment protocols for animals.

## Supplementary Material

Animal managment file; Biology letters data

## Data accessibility

The data is accessible in the CSIRO data access portal: http://doi.org/10.4225/08/573CEDB897A0C.
