# Supplementary material for: Attention bias to threat indicates anxiety differences in sheep
Source: Biol Lett. 2016 Jun;12(6):20150977. doi: 10.1098/rsbl.2015.0977 (PMC4938034; doi:10.1098/rsbl.2015.0977)
Supplement: Animal managment file; Biology letters data [file rsbl20150977supp1.docx]

**Supplementary material**

**Allocation to treatments**

For allocation of the sheep to treatments, all sheep were pooled and allocated at random to a treatment group while balancing for body weight and training group.

See Table below for the body weight average and SE of the three treatments.

| Group | Body weight | SE |
| --- | --- | --- |
| 1 | 40.0 | 0.85 |
| 2 | 40.4 | 0.82 |
| 3 | 40.9 | 0.84 |
